# Natalizumab Treatment Modulates Peroxisome Proliferator-Activated Receptors Expression in Women with Multiple Sclerosis

**DOI:** 10.1155/2016/5716415

**Published:** 2016-12-18

**Authors:** Véronique Ferret-Sena, Alexandra Maia e Silva, Armando Sena, Inês Cavaleiro, José Vale, Bruno Derudas, Giulia Chinetti-Gbaguidi, Bart Staels

**Affiliations:** ^1^Interdisciplinary Centre of Research Egas Moniz (CiiEM), Caparica, Portugal; ^2^Neurology Service, Hospital dos Capuchos, Centro Hospitalar Lisboa Central, Lisboa, Portugal; ^3^Department of Neurology, Hospital Beatriz Angelo, Loures, Portugal; ^4^University of Lille, Inserm, CHU Lille, Institut Pasteur de Lille, Lille, France; ^5^University of Côte d'Azur, CHU, CNRS, Inserm, IRCAN, Nice, France

## Abstract

Peroxisome Proliferator-Activated Receptors (PPAR) are transcription factors suggested to be involved in inflammatory lesions of autoimmune encephalomyelitis and multiple sclerosis (MS). Our objective was to assess whether Natalizumab (NTZ) therapy is associated with alterations of PPAR expression in MS patients. We analyzed gene expression of PPAR in peripheral blood mononuclear cells (PBMC) as well as blood inflammatory markers in women with MS previously medicated with first-line immunomodulators (baseline) and after NTZ therapy. No differences in PPAR*α*, PPAR*β*/*δ*, PPAR*γ*, and CD36 mRNA expression were found in PBMC between patients under baseline and healthy controls. At three months, NTZ increased PPAR*β*/*δ* mRNA (*p* = 0.009) in comparison to baseline, while mRNA expression of PPAR*γ* and CD36 (a well-known PPAR target gene) was lower in comparison to healthy controls (*p* = 0.026 and *p* = 0.028, resp.). Although these trends of alterations remain after six months of therapy, the results were not statistically significant. Osteopontin levels were elevated in patients (*p* = 0.002) and did not change during the follow-up period of NTZ treatment. These results suggest that PPAR-mediated processes may contribute to the mechanisms of action of NTZ therapy.

## 1. Introduction

Multiple sclerosis (MS) is a demyelinating and neurodegenerative disease of the central nervous system. It is generally accepted that migration of autoreactive T cells and monocytes across the blood-brain barrier (BBB) is of critical importance for the pathogenesis of the disease. Peroxisome Proliferator-Activated Receptors (PPAR) are transcription factors involved in metabolic and immune processes [1] and regulate T cell-mediated autoimmunity and severity of experimental autoimmune encephalomyelitis (EAE), an animal model of multiple sclerosis (MS) [2–4]. In MS patients, peripheral blood mononuclear cells (PBMC) exhibit decreased PPAR*γ* expression inversely correlated with disease activity and PPAR*γ* agonists may have beneficial effects [4–6]. Some studies have suggested that PPAR*α* [2] and PPAR*β*/*δ* [7] specific agonists should also be considered as possible therapeutic strategies for this disorder.

In the present exploratory study we analyzed patients treated with Natalizumab (NTZ), a humanized monoclonal antibody against *α*4 integrin molecules that inhibits transmigration of leukocytes to the CNS and induces complex alterations of immune functions in peripheral circulation [8–10]. However, the mechanisms underlying the beneficial effects and potential adverse events of this treatment in MS are not fully understood. Based on the literature, we hypothesized that NTZ therapy could change PPAR and CD36 gene expression in PBMC. CD36 is an innate immune receptor expressed in endothelial cells and microglia/macrophages upregulated by PPAR*γ* [1]. Blood levels of metalloproteinase-9 (MMP-9), neopterin, and osteopontin (OPN) were also measured, since their expression may be modulated by PPAR [1] and MS therapies [8, 11, 12].

## 2. Material and Methods

### 2.1. Patients

Twelve female patients, with relapsing-remitting MS (RRMS) and scheduled to start treatment with NTZ, were recruited from two MS clinical centers in Lisbon (Portugal). The mean age of these patients was 43 years (SD: 12); the mean duration of the disease 11.6 years (SD: 8.8); the mean expanded disability status scale is 4.1 (SD: 1.9); and the annualized relapse rate is 2.9 (SD: 1.7). The mean annualized relapse rate was calculated on the basis of the number of relapses occurring in each subject the previous two years under first-line immunomodulator treatment. Eleven patients received interferon beta-1a (Rebif, 44 *μ*g s.c.) 3 times weekly or interferon-1b (Betaferon, 250 *μ*g s.c.) every other day. One patient was medicated with glatiramer acetate injections. Blood samples were collected from these patients after a one-week washout period before starting treatment with 300 mg NTZ intravenously once monthly (baseline). At sampling, no patient was suffering from a relapse nor taking lipid-lowering agents and none had been treated with steroids for at least 1 month. Blood samples were also obtained at three and six months after switching therapy, just before the infusion of NTZ. During this study period, no patient suffered from a relapse or was medicated with corticosteroids. All patients and nine age-matched female healthy controls signed an informed consent. The local Ethics Committee approved this study.

### 2.2. RNA Extraction and PCR Analysis

Blood was processed immediately after venipuncture and PBMC were collected by a lymphocyte separation medium gradient. Purification of mRNA was processed using QIAamp RNA Blood Mini Kit (Qiagen), according to the manufacturer' protocol. PPAR*α*, PPAR*β*/*δ*, PPAR*γ*, and CD36 mRNA expression in PBMC was evaluated by quantitative RT-PCR. RNA was reverse-transcribed using random hexamer primers and Superscript reverse transcriptase (Life Technologies, France) and cDNAs were quantified either by Brilliant III Ultra-Fast* SYBRGreen *using specific oligonucleotides (for PPAR*γ*, CD36 and cyclophilin) or by Kit Brilliant Multiplex QPCR Master Mix Agilent to simultaneously detect the expression of PPAR*α*, PPAR*β*/*δ*, and cyclophilin on an Mx3000 apparatus (Stratagene, La Jolla, CA) (see Supplementary Information available online at http://dx.doi.org/10.1155/2016/5716415 for primers and probes used). The relative expression of each gene was calculated by the ΔCt method, where ΔCt is the value obtained by subtracting the Ct (threshold cycle) value of cyclophilin mRNA from the Ct value of the target gene. The amount of target relative to the cyclophilin mRNA was expressed as 2^−(ΔCt)^.

### 2.3. Biochemistry Assays

Plasma and serum were collected from the same samples and stored at −80°C until use. Commercially available enzyme-linked immunosorbent assays (ELISA) were used for measurement of MMP-9, OPN (Quantikine ELISA Kits, R&D Systems Europe, Abingdon, UK), and neopterin (ELISA Kit, IBL, Hamburg, Germany).

### 2.4. Statistical Analysis

Expression of PPAR*α*, PPAR*β*/*δ*, PPAR*γ*, and CD36 mRNA and inflammatory marker concentrations were compared between patients and healthy controls using two-sample *t*-tests. The change from baseline in these parameters at three and six months on NTZ therapy was analyzed using one-sample *t*-tests. The correlations between the changes in PPAR expression and the changes in inflammatory mediators were carried out using Pearson's correlation coefficient. A *p* value < 0.05 was considered statistically significant.

## 3. Results

**Figure 1:**
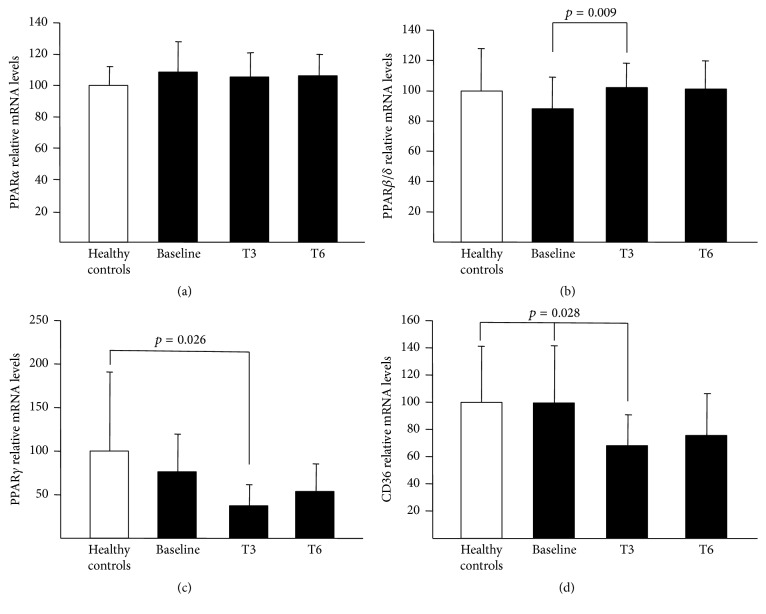
Expression of PPAR*α*, PPAR*β*/*δ*, PPAR*γ*, and CD36 in PBMC of healthy controls and patients. PBMC were isolated from healthy controls and patients at baseline and after 3 (T3) or 6 months (T6) of treatment with NTZ. mRNA levels of PPAR*α* (a), PPAR*β*/*δ* (b), PPAR*γ* (c), and CD36 (d) were measured by Q-PCR. The relative expression of each gene was calculated as described above, normalized to cyclophilin mRNA, and expressed as means ± SD relative to healthy controls set at 100.

The results concerning PPAR and CD36 mRNA in the PBMC of the studied population are presented in Figure 1. No differences in PPAR*α*, PPAR*β*/*δ*, PPAR*γ*, and CD36 mRNA expression between patients under baseline treatment and healthy controls were found. At three months on NTZ, patients had higher PPAR*β*/*δ* mRNA expression in comparison to baseline (mean difference 14.5 (95% CI: 4.4, 24.6), *p* = 0.009). In addition, NTZ treated patients had lower PPAR*γ* (difference in means −64 (95% CI: −120, −9), *p* = 0.026) and CD36 (difference in means −32 (95% CI: −60, −4), *p* = 0.028) mRNA expression at three months than normal controls. CD36 level was also lower in comparison to baseline (mean difference −32 (95% CI: −60, −4), *p* = 0.028). Although this trend of alterations remained after six months on NTZ therapy the results were not statistically significant. In contrast, this treatment did not change PPAR*α* gene expression in PBMC.

Plasma concentrations of inflammatory markers are presented in Table 1. No statistically significant differences in MMP-9 protein levels between patients and healthy controls were found. Patients under baseline had higher neopterin levels than healthy controls (difference in means 3.9 (95% CI: 0.4, 7.3), *p* = 0.029). NTZ therapy decreased neopterin to normal levels. Patients had higher OPN levels than healthy controls under baseline (difference in means 53 (95% CI: 23, 84), *p* = 0.002) and at three months (difference in means 30 (95% CI: 10, 50), *p* = 0.006) and six months (difference in means 33 (95% CI: 10, 56), *p* = 0.007) on NTZ therapy. No statistically significant correlations between the changes in PPAR expression and the changes of inflammatory mediators were observed (data not shown). These results remain unchanged whether the patient who received glatiramer acetate treatment was excluded from the analysis.

## 4. Discussion

This exploratory study suggests that NTZ induces selective alterations of PPAR*β*/*δ* and PPAR*γ* gene expression in PBMC of women with MS. This treatment is associated with peripheral sequestration of activated T cells and increased production of proinflammatory cytokines in the blood [8–10]. Inflammatory stimulation decreases PPAR*γ* promoter activity and gene transcription and PPAR*γ* agonists are anti-inflammatory and able to upregulate CD36 expression [1, 4, 5]. Therefore, the induction of systemic inflammation by NTZ could explain a decrease of PPAR*γ* and CD36 gene expression in the PBMC of patients. Importantly, systemic inflammatory activity has been linked to the beneficial effects of NTZ in reducing biomarkers of intrathecal inflammation [8, 9]. It is well accepted that NTZ blocks *α*4*β*1 (VLA-4) integrin-mediated leukocyte transmigration to the CNS [8–10]. In this regard, it is interesting that PPAR*γ* may regulate the expression of *β*1 integrin [13]. Moreover, in MS patients free of therapy, a pronounced elevation of PPAR*γ* levels in the cerebrospinal fluid (CSF) was associated with increased intrathecal inflammation [14]. Overall, these data suggest that PPAR*γ*-mediated processes may contribute to the mechanism of action of NTZ. In contrast, PPAR*β*/*δ* gene expression increased by this drug. PPAR*β*/*δ* has a complex role in immune regulation. Although PPAR*β*/*δ* agonists have strong anti-inflammatory effects, they may also induce some immune stimulatory components [15]. In experimental models, PPAR*β*/*δ* expression mediates distinctive mechanisms in suppressing CNS autoimmunity [3] and promoting myelination [7]. Therefore, the present results could indicate a link between PPAR*β*/*δ* upregulation and the protective effects of NTZ. It is remarkable that PPAR*α* mRNA levels were unchanged in our cohort of MS women. In fact, PPAR*α* expression was shown to modulate the production of proinflammatory cytokines and the development of EAE in males but not in females [2]. These findings suggest that it would be important to analyze whether PPAR*α* also modulate gender-related differences in the mechanisms of action of NTZ therapy.

It was not unexpected that plasma neopterin levels increased at baseline, since most patients have been medicated with interferon beta. The mechanism of action of this treatment is known to increase this inflammatory marker of macrophage activation [11]. Notably, early in the course of NTZ therapy, plasma neopterin decreased to normal levels. As reported in most studies, plasma OPN was increased in our patients previously medicated with immunomodulators [12, 16]. Nevertheless, in contrast to neopterin, OPN levels were not significantly changed during the first six months on NTZ therapy. In a recent study, NTZ decreased OPN levels only after 12 months of treatment in correlation with an improvement of cognitive functions [12]. In this regard, it is of potential relevance that PPAR*γ* and PPAR*α* agonists have inhibitory effects on OPN gene expression [17, 18]. The systemic profile of T cells activation and cytokine production induced by NTZ treatment has been shown to be time-dependent and may change differently in single subjects [9, 10]. A major limitation of the present results concerns the small size of the studied population and the short follow-up period of treatment. Therefore, they do not exclude a role of PPAR in modulating patients' response to this therapy. This should be tested in a larger sample cohort under a longer period of treatment and including other cytokine measurements and imaging data. A major concern associated with the continuation of NTZ therapy regards the increased risk for latent virus-infection activation, including the occurrence of progressive multifocal leukoencephalopathy. In the present context, it is to note that plasma OPN is especially increased in HIV-infected patient displaying cognitive complains [19] and that PPAR*γ* and PPAR*α* agonists may protect against HIV-induced inflammatory responses [20].

In conclusion, our findings suggest that NTZ therapy induces selective alterations of PPAR-mediated processes in circulating immune cells. These results need to be confirmed in a larger cohort of patients and longer follow-up periods of treatment. Along the reviewed data, they suggest that PPAR should be considered as potential useful biomarkers of MS patient response to NTZ therapy.

## Supplementary Material

Primers and probes used.

## Figures and Tables

**Table 1:** Inflammatory markers.

Markers	Healthy controls	MS patients						
		Baseline treatment		Natalizumab				
			*p*	3 months	*p* ^*∗*^	*p* ^*∗∗*^	6 months	*p* ^*∗∗∗*^
MMP-9	636.3 (349.9)	565.7 (280.7)	0.613	474.7 (214.1)	0.205	0.446	467.2 (209.2)	0.385
Neopterin	5.7 (1.4)	9.6 (4.8)	0.029	6.6 (1.4)	0.144	0.033	6.0 (0.5)	0.018
Osteopontin	51.1 (18)	104.1 (40.6)	0.002	81 (24.5)	0.006	0.073	84.2 (29.1)	0.225

Values shown are mean (± SD) ng/mL.

*p* —comparison between patients at baseline and healthy controls.

*p* ^*∗*^—comparison between patients at three months on Natalizumab therapy and healthy controls.

*p* ^*∗∗*^—comparison between patients at baseline and at three months on Natalizumab therapy.

*p* ^*∗∗∗*^—comparison between patients at baseline and at six months of Natalizumab therapy.
