# Supplementary material for: Natalizumab Treatment Modulates Peroxisome Proliferator-Activated Receptors Expression in Women with Multiple Sclerosis
Source: PPAR Res. 2016 Dec 18;2016:5716415. doi: 10.1155/2016/5716415 (PMC5203914; doi:10.1155/2016/5716415)
Supplement: Supplementary file 1 — Primers and probes used. [file 5716415.f1.docx]

**Supplementary Information: Primers and probes used**

| **Method** | **Gene** | **Reverse primer** | **Forward primer** | **Probe** |
| --- | --- | --- | --- | --- |
| Multiplex | PPARα | 5’-CGG CGA GGA TAG TTC TGG AAG C-3’ | 5’-GCT CGA AGC TGG TGA AAG CGT -3’ | 5’-FAM-CGT GAT GAC CGA GCC ATC TGA GCC A-BHQ1-3 |
|  | PPARβ/δ | 5’-ATT CAT TGC GGC CAT CAT TCT G-3’ | 5’-TGG TGT CCT GGA TAG CCT CCA C-3’ | 5’-Cy5-AGA CCG GCC AGG CCT CAT GAA CGT T-DDQ2-3’ |
|  | cyclophilin | 5’-TGG TCA ACC CCA CCG TGT TC-3’ | 5’-TGC AAA CAG CTC AAA GGA GAC G-3’ | 5’-YakimaYellow-TTG CCG TCG ACG GCG AGC CCT T-BHQ1-3’ |
| Sybrgreen | PPARγ | 5’-AGT CCT CAC AGC TGT TTG CCA AGC-3’ | 5’-GAG CGG GTG AAG ACT CAT GTC TGT C-3’ |  |
|  | CD36 | 5’-TCA GCA AAT GCA AAG AAG GGA GAC-3’ | 5’-GGT TGA CCT GCA GCC GTT TTG-3’ |  |
|  | cyclophilin | 5’-GCA TAC GGG TCC TGG CAT CTT GTC C-3’ | 5’-ATG GTG ATC TTC TTG CTG GTC TTG C-3’ |  |
